# Detection of *Trichomonas vaginalis* in prostate tissue and serostatus in patients with asymptomatic benign prostatic hyperplasia

**DOI:** 10.1186/s12879-016-1843-1

**Published:** 2016-09-23

**Authors:** Jamshaid Iqbal, Jumanah Al-Rashed, Elijah O. Kehinde

**Affiliations:** 1 Department of Medical Microbiology, Faculty of Medicine, Kuwait University, PO Box: 24923, Safat, 13110 Kuwait; 2 Department of Surgery (Division of Urology), Faculty of Medicine, Kuwait University, PO Box: 24923, Safat, 13110 Kuwait

**Keywords:** Benign prostate hyperplasia, *Trichomonas vaginalis*, Prostate tissue, Serostatus

## Abstract

**Background:**

Despite a worldwide common and progressive nature of benign prostate hyperplasia (BPH) in older men, no association has been observed between a causative pathogen and other etiology so far.

**Methods:**

In this study, we investigated a causative association of *Trichomonas vaginalis,* a flagellate protozoan parasite, in 171 BPH cases presenting without symptoms of prostatitis at a surgical outpatient clinic in Kuwait. We detected *T. vaginalis* DNA by polymerase chain reaction (PCR) and *T. vaginalis* antigen by immunocytochemistry (ICC) in the prostate tissue of these cases. A total of 171 age-matched controls with no urinary tract symptoms were also included in the study. A detailed information regarding the sexual history and sexually transmitted infections (STIs) was enquired from all the enrolled subjects.

**Results:**

We detected *T. vaginalis* DNA and *T. vaginalis* antigen in 42 (24.6 %) and 37 (21.6 %) of the 171 BPH cases respectively in their prostate tissue. Both these assays showed a very good agreement and statistically no significant difference in their sensitivities and specificities. A relatively higher seropositivity rate for antibodies to *T. vaginalis* was detected in BPH cases (53 of 171 cases, 31.0 %) than in the control group (26.9 %) [p: 0.19] and both were higher than in earlier reports but no significant association was observed between BPH and *T. vaginalis* serostatus. However, a greater proportion of seroreactive BPH cases had high IgG2 antibody absorbance score than in the control group (p:0.000). Furthermore, no significant association was observed between *T. vaginalis* seropositivity and presence of *T. vaginalis* DNA in the prostate tissue.

**Conclusions:**

Our study documents *T. vaginalis* DNA and *T. vaginalis* antigen in 24.6 and 21.6 % respectively in the prostate tissue of the BPH cases. We also detected a relatively higher seropositivity rate for antibodies to *T. vaginalis* both in the BPH cases and in normal control group, 31 and 26.9 % respectively but no significant association was observed between BPH and *T. vaginalis* serostatus or presence of *T. vaginalis* DNA in the prostate tissue. Further epidemiological and case-controlled studies are needed to focus on local response to chronic asymptomatic retention of *T. vaginalis* in prostate tissue in the development of benign prostate hyperplasia.

**Electronic supplementary material:**

The online version of this article (doi:10.1186/s12879-016-1843-1) contains supplementary material, which is available to authorized users.

## Background

Benign prostatic hyperplasia (BPH) and prostate cancer (PCa) represent the most common urologic diseases among the elderly males resulting in more than 2 million visits per year; 8 % of the total urologic visits and 1 % of family physician visits in the USA [1–3]. Its prevalence among men at risk is believed to be between 5 and 8 % [4, 5]. BPH and PCa are considered chronic diseases, with early initiation and slow progression. Despite extensive research, the pathophysiology of BPH and PCa is not yet completely understood however, several observational and case-control studies have documented the role of sexually transmitted infections (STIs) in chronic inflammation as an important factor in BPH development and progression [6–8]. Several sexually transmitted agents such as *Neisseria gonorrhoeae, Chlamydia trachomatis* and *Trichomonas vaginalis (T. vaginalis)* are known to cause chronic inflammation within the prostate gland’s tissue [9]. However, a recent large retrospective and prospective study did not support associations of several known STIs with BPH with the exception of *T. vaginalis* infection that showed modest association with BPH [10]. *T. vaginalis* is a common parasitic sexually transmitted infection, with an estimated 174 million annual infections globally. Studies have shown that *T. vaginalis* can be associated with asymptomatic infections in 50–75 % of infected men and a number of observations support an association between *T. vaginalis* and prostatitis [11, 12]. As such, chronic prostatic infection with *T. vaginalis* may initiate an inflammatory response that could increase the risk of developing BPH and PCa. The objective of the present study was to investigate the potential association between *T. vaginalis* infection and BPH by detecting *T. vaginalis* DNA and antigen in the prostate tissue and *T. vaginalis* serostatus in patients with BPH without clinical symptoms of prostatitis.

## Methods

### Patient selection

A total of 177 BPH cases aged >60 years of age without clinical symptoms of prostatitis reporting at the Urological Clinic, Mubarak Al-Kabir Teaching Hospital, Kuwait were registered and treated for BPH by transurethral resection of the prostate (TURP) during the period June 2013-December 2014. All enrolled cases were given a code number to preserve their confidentiality. From each of the enrolled cases, 5–6 small biopsy specimens were taken from the prostate for histopathology routine examination to confirm the diagnosis of BPH and 5 ml. blood to assess *T. vaginalis* serostatus. The biopsy specimens were also processed to detect *T. vaginalis* DNA and antigen in the tissue. The tissue was also processed to detect other relevant bacterial and viral pathogens by multiplex PCR and culture. A detailed information regarding the sexual history and sexually transmitted infections (STIs) was enquired from all the enrolled subjects. When available, their hospital records were also screened for this information.

We also included 177 control subjects from the same population group who had provided blood at the outpatient clinic for conditions other than prostate and/or urinary tract infection. For statistical efficiency, control subjects were individually matched to BPH cases by age.

### Polymerase chain reaction (PCR) for detection of *T. vaginalis* DNA in prostate tissue

For detection of *T. vaginalis*, the prostate tissue was processed and the DNA extracted as described earlier [13, 14]. Briefly, the tissue samples were cultured in TYI S 33 medium with 10 % bovine serum albumin at 37 °C for four days under anerobic conditions. The presence of *T. vaginalis* parasite was confirmed by light microscopy of the pellet after centrifugation of the culture supernatant. *T. vaginalis*-specific PCR was performed directly on the prostate tissue specimens and the DNA was extracted using QIAamp DNA Mini kit (Qiagen) following manufacturer’s instructions. A set of primers targeting a conserved region of the beta-tubulin genes of *T. vaginalis* (*btub1-2*, and −*3*) was used for amplification; for ***BTUB 9:*** 5′ CAT TGA TAA CGA AGC TCT TTA CGA T 3′ (positions 850–874), and for ***BTUB 2:*** 5′ GCA TGT TGT GCC GGA CAT AAC CAT 3′ (positions 961–938) [13, 14]. The performance of beta-tubulin primers BTUB9 and BTUB2 was earlier evaluated in a pilot study to screen pregnant women for STDs including *T. vaginalis* at the maternity clinic in Kuwait during 2012–2013 using a series of positive controls.

A positive (ATCC 30236) and a negative (distilled water) control was used with each run. The DNA was amplified by PCR using an automated thermocycler (Perkin-Elmer Cetus, Norwalk, Conn.) using the standard final PCR reaction mixture (50 μl). A single predicted 112 bp PCR product was visualized on a 2 % agarose gel stained with ethidium bromide. All PCR positives were confirmed by sequence analysis of the BTUB PCR product using a 3100-*Avant* Genetic analyzer (Applied Biosciences) and comparing data with Genebank as reported earlier [14].

### Immunocytochemistry (ICC) of fixed prostate tissue to detect *Trichomonas vaginalis* parasite antigen (TV Ag)

The fresh biopsies were fixed in formalin, processed and embedded in paraffin wax and a 4–5 μm sections were cut. Staining was done after deparaffinization and rehydration by graded alcohols. Endogenous peroxidase activity was blocked using 0.3 % hydrogen peroxide in Tris-buffered saline. For antigen retrieval the sections were treated with 10 mmol/l citrate buffer (pH 6.1) in microwave for 5 min and then incubated with the mouse primary monoclonal anti-*Trichomonas vaginalis* antibody (MYBioSource, San Diego, USA). Avidin-biotin complex (ABC) method was used for antigens visualization. Briefly, EnVision Flex Dual Link horseradish peroxidase/DAB visualization system (Dako) was used and counterstained with haematoxylin. The specimen was considered positive if either nuclear only staining or nuclear and cytoplasmic staining was present. Positive control slides with *Trichomonas vaginalis* parasites were used for each run of samples.

### Detection of *T. vaginalis* serostatus

*T. vaginalis* antibody serostatus (IgG, IgG subtypes) was measured by using an in-house indirect ELISA that detects antibodies against purified, recombinant α-actinin protein from *T. vaginalis* parasite (strain C-1:NIH) as described earlier [15] with slight modifications. Briefly, the recombinant α-actinin protein was purified from the harvested *Escherichia coli* colony containing the recombinant plasmid wit α-actinin gene. Optimum dilution of the recombinant protein, serum and antihuman conjugates used in ELISA to screen for serostatus were determined by a checker-board titration. A panel of five known positive specimens from confirmed *T. vaginalis* cases and negative pooled specimens from uninfected individuals were used in random order with each run of the assay to determine absorbance score. Briefly, 100 μl of paired plasma samples from the cases and controls diluted at 1:200 in phosphate-buffered-saline-Tween 20 containing 5 % skim milk was added into microtiter wells coated with recombinant α-actinin protein overnight. All specimen were used in duplicate in optimum dilutions as determined above (IgG; 1:200, IgG subtypes; 1:40) and inferences were based on the mean of duplicate values. The horseradish peroxidase-labelled antihuman IgG and the biotin-labelled antihuman IgG1/IgG2/IgG3/IgG4 (Sigma, St. Louis, MO, USA) were used at the calculated optimum dilution in PBS—Tween 20—BSA. The working dilution of HRP-labelled antihuman IgG was determined at 1:5000 and for IgG1/IgG2/IgG3/IgG4 at 1:1000, 1:5000, 1:2500 and 1:5000, respectively. The plates were incubated for 1 h at 37 °C for 30 min followed by incubation with the substrate H_2_O_2_-OPD in citrate buffer. The reaction as stopped with H_2_SO_4_. The cut-off points for seropositivity were determined by obtaining positive to negative (P/N) ratios of the mean of duplicate optical densities (ODs) ± 2 standard deviations (SD) values of the positive and negative control specimens as described earlier [15, 16]. The absorbance score was then determined by comparing the P/N values with ODs of the known positive control specimens. Samples with absorbance score of 1 with mean OD value of >0.70 were considered positive for *T. vaginalis* infection.

### Ethics statement

The present study was reviewed and approved by the Faculty of Medicine, Kuwait University and Kuwait Institute for Medical Specialization (KIMS) joint Research Ethics Committee. The Committee reviews experimental protocols involving human subjects and/or animal as per international standardized ethical guidelines. All patients gave their informed consent to their prostate tissue and blood sample for testing and they were informed of their study-related test results.

### Statistical analysis

Statistical analysis were performed using SPSS 12.0.1 (SPSS Inc.). We used One way ANOVA and Post Hoc tests to analyze variables (total prostate specific antigen and antibody absorbance means, medians and proportions) by *t* test using independent samples. The comparative performance of *T. vaginalis* antigen test and PCR assay was done by McNemar test. To explore potential associations of *T. vaginalis* serostatus and BPH, we used conditional logistic regression to analyze potential BPH risk according to *T. vaginalis* antibody serostatus. Odds raios (ORs) and 95 % confidence intervals (CIs) were estimated by comparing average optical density values and positive/negative values of *T. vaginalis* seropositive and -negative cases. Differences were considered significant with *p* <0.05.

## Results

The prostate tissue from all 177 BPH cases was processed for PCR analysis using a set of BTUB 9/2 primer set targeting a well-conserved region in the beta-tubulin genes of *T. vaginalis* (*btub1-2*, and −*3*) which amplified a single predicted 112 bp PCR product visualized on gel electrophoresis. We detected *T. vaginalis* DNA by PCR in 43 of 177 (24.3 %) cases. Six of the BPH cases who had characteristic BPH findings on routine histological examination were also shown to have additional incidental prostate cancer (iCaP), of which one was also positive for *T. vaginalis* DNA. The iCaP cases were removed from further testing and statistical analysis. Thus, *T. vaginalis* DNA was detected in 42 of 171 (24.6 %) BPH cases in prostate tissue specimens by PCR (Table 1). All PCR positives were confirmed by sequence analysis of the BTUB PCR product using a 3100-*Avant* Genetic analyzer and showed highest similarity to *Btub1* (data not shown). The mean age of BPH cases positive for *T. vaginalis* DNA was 65. 6 years (SD = ±5.3 years) and the DNA- negative cases was aged 65.8 years (SD = ±2.3 years) (Table 1).

**Table 1:** Trichomonas vaginalis infection detection status by PCR and age of BPH patients

Diagnosis	*T. vaginalis positive* (by PCR)		*T. vaginalis negative* (by PCR)	
	*N (%)*	Mean age ± SD	*N (%)*	Mean age ± SD
BPH and iCaP	43/177 (24.3)	65.7 ± 4.2	134/177 (75.7)	65.8 ± 5.1
BPH only	42/171 (24.6)	65.6 ± 5.3	129/171 (75.4)	65.8 ± 2.3

*BPH* benign prostatic hyperplasia, *iCaP* incidental prostate cancer

The *T. vaginalis* culture was positive only in 6 of 171 tissue specimens. The *T. vaginalis* antigen in prostate tissue was detected by immunocytochemical (ICC) staining using purified primary monoclonal anti-*Trichomonas vaginalis* antibody. TV Ag assay was used to detect TV infection in the prostate tissue in this study in addition to TV-DNA test to evaluate its performance using TV-DNA test as the standard test. TV Ag was detected in 37 of 171 specimens (21.6 %); all of which were also positive for *T. vaginalis* DNA by PCR however, 5 of the PCR positive specimens were negative for *T. vaginalis* antigen (Table 2). Using PCR for detection of *T. vaginalis* infection as the standard test, statistical analysis using McNemar test for Kappa agreement showed very good strength between the PCR and *T. vaginalis* antigen test and statistically no significant difference in their sensitivities (p: 0.25); antigen sensitivity: 89.29 %, 95%CI = 71.77–97.73 %; antigen specificity: 100 %, 95%CI = 95.85–100 %) (Table 2). No relevant co-infection of bacterial (syphilis, gonorrhea and chlamydia) and viral pathogens (herpes simplex virus and human papillomavirus) was detected in prostate tissue screened simultaneously for *T. vaginalis* by PCR (data not shown). A very limited information was available on the sexual history and STIs from all the enrolled cases and the controls and from their hospital records, where available. The mean age of our study group was >65 years and only 3 BPH cases and 5 controls admitted having multiple sexual partners in the past but they claimed always practicing safe sex.

**Table 2:** Comparative performance of T. vaginalis antigen test by immunocytochemistry assay and PCR assay to detect T. vaginalis DNA in the prostate tissues from BPH cases

		PCR		TOTAL
		+	−	
Ag. test	+	37	0	37
	−	5	129	134
	TOTAL	42	129	171

Ag vs. PCR• Kappa agreement: Value = 0.93; SE = 0.04; 95 % CI = 0.84–1.0; Strength = very good• McNemar test: *p*-value = 0.25, not statistically significant difference in tests’ sensitivities• antigen sensitivity: 89.29 %, 95%CI = 71.77–97.73 %; antigen specificity: 100 %, 95%CI = 95.85–100 %)

*SE* standard error, *CI* confidence interval

*T. vaginalis* antibody infection or serostatus was determined in all BPH cases and controls by detecting IgG and IgG subtype levels against purified recombinant α-actinin proteins from *T. vaginalis.* The cut off values for seropositivity or *T. vaginalis* antibody infection was determined by obtaining P/N ratios and absorbance score as described earlier in materials and methods. The cut off value for seropositive status was determined as mean OD value of 0.70 for the IgG. A total of 53 of 171 (31.0 %) BPH cases and 46 of 171 (26.9 %) control cases were seropositive for *T. vaginalis* infection (p: 0.19; Table 3). In general, *T. vaginalis* antibody mean absorbance values for IgGs were not significantly different in BPH or the control group, 0.569 vs 0.561 respectively (p:0.663; Table 3). However, a greater proportion of seroreactive BPH cases had high IgG2 subtype absorbance score than in the control group with the mean absorbance score of 0.202 vs 0.158 respectively (p:0.000; Table 3). Additional data on BPH PCR and seroreactivity of BPH and control cases and their statistical analysis is presented in the additional files.﻿ A summary of mean OD values of IgG and IgG sub-types in BPH and control cases is presented in Table 3.

**Table 3:** T. vaginalis a-actin IgG and IgG-subtype antibody levels in 171 benign prostate hyperplasia cases and the control group

	BPH cases	Control group	*p* ^a^
*T. vaginalis antibody serostatus. No. (%)*	53 (31.0 %)	49 (28.7 %)	0.19
Mean OD^a^			
Total IgGs	0.569	0.561	0.663
Median OD			
Total IgGs	0.592	0.567	
Mean OD			
IgG1	0.086	0.088	0.758
IgG2	0.202	0.158	0.000^a^
IgG3	0.159	0.153	0.374
IgG4	0.113	0.112	0.936

^a^ optical density

## Discussion

Despite a worldwide common and progressive nature of benign prostate hyperplasia in older men, no association has been observed between a causative pathogen and other etiology so far. In this study, we investigated a causative association of *T. vaginalis* in men >65 years of age with BPH by detecting *T. vaginalis* DNA and antigen in the prostate tissue of BPH cases presenting without symptoms of prostatitis at a surgical outpatient clinic in Kuwait. We detected *T. vaginalis* DNA and *T. vaginalis* antigen in 24.6 and 21.6 % in the prostate tissue of the BPH cases respectively.

Although several previous case-control studies have observed positive associations between symptomatic STI, gonorrhea and syphilis and prostate cancer, the information on association of asymptomatic STI is limited and more so for BPH cases with the exception of *T. vaginalis* infection that showed modest association with BPH cases [14]. Our finding for *T. vaginalis* infection in BPH cases is consistent with earlier studies that reported a high prevalence of *T. vaginalis* DNA in prostate tissue (34 %) from men who underwent TURP for BPH [14] and the urine of patients with chronic prostatitis (21.2 %) [17]. The detection rate of *T. vaginalis* infection in men has been shown to vary according to geographical location, presence or absence of symptoms, age, presence or absence of *T. vaginalis* infection in sexual partners and diagnostic methods [18, 19]. We used PCR assay with BTUB primer pair which is shown to have increased sensitivity and specificity [14, 19, 20]. The specificity of PCR data was further complemented by detection of *T. vaginalis* antigen in the prostate tissue with primary monoclonal anti-*Trichomonas vaginalis* antibody by immunocytochemistry assay. Both TV-DNA and TV Ag assays were used to measure TV infection. While TV-DNA test has been used in earlier studies to detect TV infection, we also included TV Ag assay in this study to evaluate its performance in detecting TV infection in the prostate tissue. Both these assays showed very good agreement and no statistically significant difference in their sensitivities and specificities (antigen sensitivity: 89.29, antigen specificity; 100 %; p:0.25).

In our study, the mean age of all registered cases was 65.6 ± 5.3 years but no information was available on the *T. vaginalis* infection status of their sexual partners. Very limited information regarding the sexual history and STIs was available from all the enrolled subjects. When available, their hospital records were also screened for this information. The Kuwaiti population is a very conservative society esp. with regards to extra- marital sexual activities and as such, STI data registry is very limited at the STD clinics. The mean age of our study group was 65 years and as expected, only 3 BPH cases and 5 controls admitted having multiple sexual partners in the past but they claimed always practicing safe sex. The most probable and common route of transmission is sexual however, since the majority of infections in men are asymptomatic and they don’t take treatment when their sexual partners (spouses) are diagnosed with *T. vaginalis* so the men may be carrying this infection unnoticed. We suspect that these self-reported rates of trichomoniasis were likely underreported the true infection among the cases and controls because trichomoniasis is frequently asymptomatic and infrequently diagnosed in men seeking STI care. In addition, one of the selection criteria of our cases was BPH without clinical symptoms of prostatitis to rule out pathogenic and/or non-pathogenic acute infections. As such, *T. vaginalis* seroreactivity status was used to assess a history of trichomoniasis to establish a temporal relationship between trichomonal exposure and BPH, and to detect asymptomatic infections that might otherwise not be registered by self-report.

Recently, exposure to environmental factors such as infectious agents, hormonal imbalances and dietary carcinogens were proposed to lead to injury of the prostate and to the development of chronic inflammation however, no such hypothesis was forwarded for BPH [19]. A relatively high detection rate of *T. vaginalis* DNA by PCR and trichomonas specific antigen by ICC in the prostate tissue of asymptomatic BPH cases in this report provides a strong direct evidence of intraprostatic *T. vaginalis* infection indicating chronic retention of this parasite in the prostate tissue, which may be linked to development of BPH. Recently, an in vitro evidence of inflammatory response of prostate epithelial cells to stimulation by *T. vaginalis* mediated by immune cells and cytokines supported the role of trichomonas in prostate hyperplasia [19].

In this study, the seropositivity for antibodies to *T. vaginalis* in BPH cases (31.0 %) and in the control group (26.9 %) was relatively higher in our study population than some of the earlier reports (p: 0.19) [16, 21]. A panel of five known seropositive and seronegative pooled specimens were included with each test run to determine the cut-off points and absorbance score for *T. vaginalis* infection. The differences in assay sensitivities and demographic characteristics may account for some of the variations in distribution of *T. vaginalis* seropositivity in these studies. In general, *T. vaginalis* antibody mean ODs for IgGs were not significantly different in BPH or the control group, 0.569 vs 0.561 respectively (p:0.663). However, a greater proportion of BPH cases had high IgG2 subtype OD values than in the control group with the mean ODs of 0.202 vs 0.158 respectively (p:0.000) which may be incidental and we don’t have any explanation for its biological relevance. Furthermore, no significant association was observed between seroreactivity and *T. vaginalis* DNA status in the prostate tissue (p:0.259). A number of large nested case-control studies have observed a positive association between *T. vaginalis* antibody seropositivity and prostate cancer [16, 21–23] but very little is known on seroreactivity to *T. vaginalis* in BPH cases. Recently, in a large retrospective and prospective study most of the STIs did not contribute to BPH development but a modest association of *T. vaginalis* infection with nocturia and a large prostate volume was observed [10]. A number of reports documenting serum antitrichomonal antibodies in male and female patients with acute and chronic trichomoniasis [24], and the increase in the prevalence of *T. vaginalis* with age suggested the prostate to be the reservoir for *T. vaginalis* [25, 26].

## Conclusions

In conclusion, we report an expected association of *T. vaginalis* infection in BPH cases by detecting *T. vaginalis* DNA and antigen in 24.6 and 21.6 % respectively in the prostate tissue of the BPH cases not presenting with symptoms of prostatitis. The seroprevalence of *T. vaginalis* infection was 31.0 % in BPH cases and 26.9 % in the control subjects with no significant difference in their mean absorbance values or presence of *T. vaginalis* DNA in the prostate tissue. We also conclude from this study that a large proportion of the detected trichomonas infections in men were chronic, asymptomatic infections, which, without treatment, may have progressed to the prostate tissue causing chronic prostate infection. This would induce an inflammatory response in the normal prostate that may potentially lead to hyperproliferation of prostatic epithelial cells leading to BPH. Further epidemiological and case-controlled studies are needed to focus on local response to chronic asymptomatic retention of *T. vaginalis* in prostate tissue in the development of BPH.

## Additional file

Additional file 1: Raw data and statistical analysis. All data generated or analyzed during this study on each individual benign prostate hyperplasia (BPH) and control case is presented. The data includes information on the following: -Levels of IgG subtypes (OD values), -*T vaginalis*-PCR positive and negative cases (OD values), -All statistical data analyzed during this study on IgG subtypes among BPH and control subjects. (XLSX 39 kb)

## Abbreviations

BPH: Benign prostate hyperplasia
ICC: Immunocytochemistry
PCR: Polymerase chain reaction
*T. vaginalis*: *Trichomonas vaginalis*
